# E3 Ligase Nedd4 Promotes Axon Branching by Downregulating PTEN

**DOI:** 10.1016/j.neuron.2010.01.017

**Published:** 2010-02-11

**Authors:** Jovana Drinjakovic, Hosung Jung, Douglas S. Campbell, Laure Strochlic, Asha Dwivedy, Christine E. Holt

**Affiliations:** 1 Department of Physiology, Development and Neuroscience, University of Cambridge, Downing Street, Cambridge CB2 3DY, UK

**Keywords:** DEVBIO, MOLNEURO, SIGNALING

## Abstract

Regulated protein degradation via the ubiquitin-proteasome system (UPS) plays a central role in building synaptic connections, yet little is known about either which specific UPS components are involved or UPS targets in neurons. We report that inhibiting the UPS in developing *Xenopus* retinal ganglion cells (RGCs) with a dominant-negative ubiquitin mutant decreases terminal branching in the tectum but does not affect long-range navigation to the tectum. We identify Nedd4 as a prominently expressed E3 ligase in RGC axon growth cones and show that disrupting its function severely inhibits terminal branching. We further demonstrate that PTEN, a negative regulator of the PI3K pathway, is a key downstream target of Nedd4: not only does Nedd4 regulate PTEN levels in RGC growth cones, but also, the decrease of PTEN rescues the branching defect caused by Nedd4 inhibition. Together our data suggest that Nedd4-regulated PTEN is a key regulator of terminal arborization in vivo.

## Highlights

► The Ubiquitin-Proteasome System is required for terminal branching of retinal axons ► E3 ligase Nedd4 downregulates PTEN in retinal axon growth cones ► PTEN downregulation by Nedd4 promotes terminal branching of retinal axons

## Introduction

A key aspect of the specification of neuronal connectivity is the guidance of axons to their correct synaptic partners. Axons often traverse long distances to reach their final destination by responding to molecular cues along the pathway that induce cytoskeletal rearrangements in axonal growth cones (GCs) (Guan and Rao, 2003). Upon reaching its target an axon loses its GC and instead elaborates a branched terminal arbor with which synaptic contacts are made, which is a crucial step for proper development of neuronal circuits. Indeed, both the lack of terminal axon growth (Thomas and Wyman, 1984) and excessive axonal (Smear et al., 2007) and dendritic branching (Kwon et al., 2006) have been shown to perturb behavioral responses. Similarly to axon guidance, axon branching is regulated by extracellular cues (Campbell et al., 2001, 2007; Cohen-Cory and Fraser, 1995; Dent et al., 2004; Roskies and O'Leary, 1994; Wang et al., 1999). However, unlike axon guidance, where the mechanisms by which extracellular cues induce GC turning have been fairly well characterized, little is known about the intracellular signaling pathways that regulate axon branching.

In recent years, ubiquitin-mediated protein degradation, also known as the ubiquitin proteasome system (UPS), has emerged as a cellular pathway with key roles in regulating diverse aspects of neural development, including neural connectivity (Yi and Ehlers, 2007). Proteins that are targeted for UPS-mediated degradation are tagged with polyubiquitin chains through the concerted action of several enzymes, including a ubiquitin activating enzyme (E1), a ubiquitin conjugating enzyme (E2), and a ubiquitin ligase (E3), and as such are recognized by the 26S proteasome and degraded (Glickman and Adir, 2004). Crucially, substrate recognition is mediated by the E3 ligases, and therefore the specificity of the system is principally determined by these key components (Yi and Ehlers, 2007). Our group has shown that the core UPS components, including ubiquitin, E1, and the 26S proteasome, are present in axons and GCs of *Xenopus laevis* retinal ganglion cells (RGCs), and that UPS activity is essential for RGC GC chemotropic turning in vitro (Campbell and Holt, 2001). It is still unclear, however, which E3 ligase or ligases might be involved, and which substrates are targeted for degradation in RGC axons. Nedd4 (neural precursor cell-expressed developmentally down-regulated gene 4) belongs to a HECT (homologous to E6-AP carboxyl terminus) family of E3 ligases, and it has been found to play key roles in aspects of neuronal development in invertebrates, including axon guidance and synaptogenesis in *C. elegans* (Schmitz et al., 2007; Sieburth et al., 2005), midline crossing of commissural axons in *Drosophila* (Myat et al., 2002), and postsynaptic maturation of neuromuscular junction (NMJ) in *Drosophila*. Although widely expressed in the vertebrate CNS (Kumar et al., 1997; Kumar et al., 1992), and found to regulate survival of cultured vertebrate DRG neurons (Arévalo et al., 2006), the role of Nedd4 in the assembly of vertebrate neural projections has been largely unexplored. A recent report identified the phosphatase PTEN (phosphatase and tensin homolog deleted on chromosome 10) as a Nedd4 substrate that is degraded by the UPS in cancerous cell lines (Wang et al., 2007). PTEN is an important negative regulator of the evolutionarily conserved phosphatidylinositol-3-kinase (PI3K) signaling pathway that regulates cell survival, differentiation, size, and migration. The downstream effectors of PI3K signaling, among other roles, act to regulate cytoskeletal dynamics, and thus have a profound impact on cell motility and remodeling of neuronal morphology (Cosker and Eickholt, 2007). Our goal in this study was to explore if the UPS and Nedd4, and its substrate PTEN, have a role in the development of vertebrate neural circuits by using the *Xenopus* retinotectal projection as an in vivo model system.

In this study we demonstrate that perturbing the UPS in vivo suppresses RGC axon branching in the tectum. Next, we show that the E3 ligase Nedd4 is expressed in RGC axons and regulates their branching in vivo. Finally, we show that PTEN is coexpressed with and negatively regulated by Nedd4 in RGC axons. Crucially, we show that decreasing PTEN levels in RGC axons rescues the axon branching defect caused by Nedd4 inhibition. Together, our data suggest a model wherein Nedd4 downregulates PTEN via the UPS in RGC axons upon reaching their synaptic target and thus promotes PI3K-induced cytoskeletal arrangements that bring about branch formation.

## Results

### UPS Is Required for Axon Branching

To investigate the involvement of the UPS in axon guidance and branching in vivo, we sought to inhibit protein polyubiquitination and degradation in the RGCs. Proteins destined for degradation in the 26S proteasome are tagged with polyubiquitin chains. These are assembled through a step-wise addition of a new ubiquitin group to the Lys-48 (K48) residue of the last ubiquitin added. Mutation of Lys-48 to Arg results in a dominant-negative form of ubiquitin (UbK48R) that can still be conjugated onto a substrate, but can no longer form a step in polyubiquitin chains (Finley et al., 1994). Consequently, overexpression of UbK48R mutant leads to the inhibition of polyubiquitination and has previously been used successfully to inhibit UPS-dependent protein degradation in neurons (Patrick et al., 2003).

**Figure 1:**
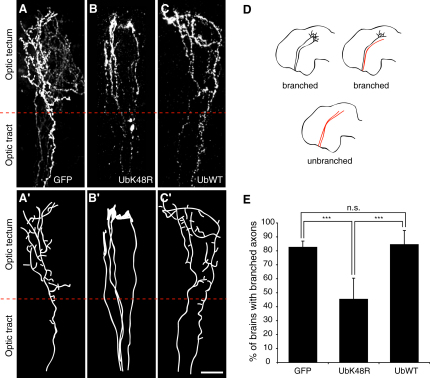
UbK48R Inhibits RGC Axon Branching in the Tectum (A–C) Lateral view of RGC axons in the optic tract and tectum expressing control GFP (A), Myc-UbK48R (B), or Myc-UbWT (C). Axon trajectories are represented in (A′)–(C′). Scale bar, 20 μm. (D) A strategy for quantifying the extent of branching from a population of brains with axons expressing a given construct. Brains that have at least one branched axon are scored as “branched” and brains with no branched axons are scored as “unbranched.” (E) Graph showing the proportions of brains with branched axons that express GFP, UbK48R, or UbWT. Numbers of brains analyzed: GFP (n = 75), UbK48R (n = 11), UbWT (n = 13).

In order to inhibit the UPS specifically in RGC axons in vivo, the Myc-tagged UbK48R cDNA was electroporated directly into the embryonic retina at the onset of axonogenesis (stage 28). From stage 28 to stage 39/40, a period of 15–24 hr, pioneering axons follow a stereotyped trajectory from the eye through the contralateral optic tract to the optic tectum, a distance of approximately 800 μm (Dingwell et al., 2000). We found that whereas UbK48R-expressing axons were able to pathfind correctly from the retina to the tectum, these axons failed to branch correctly after entering the tectum and instead retained terminal GCs (Figures 1A–1C). To quantify the extent of branching, we counted the number of electroporated brains containing branched axons for each construct tested (Figure 1D). At stage 43, the vast majority of brains with axons expressing a control membrane GFP (GFP) or wild-type ubiquitin (UbWT) had branched axons, while this number was greatly reduced in brains with UbK48R axons (Figure 1E). These results suggest that ubiquitin-mediated degradation is not essential for axon guidance over long distances, but that it is crucial for axon branching in the target area.

### Ubiquitin Ligase Nedd4 Is Expressed in RGC Axons

Having found that the UPS is required for axon branching, we asked which specific components of the UPS pathway might be involved in this process. We focused on E3 ligases because they play the central role in determining the specificity of the degradation process. Nedd4 is an E3 ubiquitin ligase that has previously been shown to affect axon guidance and synaptogenesis in *Drosophila* and *C. elegans* motor axons (Ing et al., 2007; Myat et al., 2002; Schmitz et al., 2007; Sieburth et al., 2005). We thus examined whether *Xenopus* Nedd4 might play a similar role in RGC axons.

**Figure 2:**
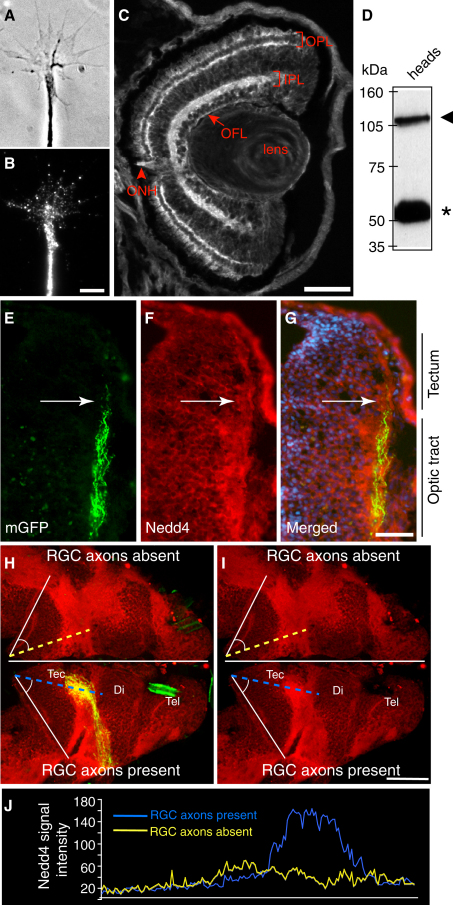
Nedd4 Is Expressed in RGC Axons (A and B) Stage 35/36 retinal explants were cultured for 24 hr and stained with an anti-Nedd4 antibody. Phase contrast image (A) of a GC-expressing Nedd4 (B) is shown. Scale bar, 5 μm. (C) Transverse section through stage 40 retina stained with an anti-Nedd4 antibody. IPL, inner plexiform layer; OPL, outer plexiform layer; OFL, optic fiber layer; ONH, optic nerve head. Scale bar, 50 μm. (D) Western blot of stage 40 head lysate probed with an anti-Nedd4 antibody. The arrowhead points to the full-length Nedd4, and the asterisk denotes a possible proteolytic fragment (Harvey et al., 1998). (E–G) Transverse section through stage 40 brain showing GFP-labeled RGC axons in the optic tract and the tectum (E); the same section stained with anti-Nedd4 is shown in (F), and merged image including DAPI (blue) is shown in (G). (H) Stage 40 brain from an embryo whose right eye was electroporated with GFP and whose left eye was removed before axon outgrowth. The brain was split along the ventral midline and flat-mounted to expose both halves of the tectum. (I) The same brain as in (H) but without the GFP signal. Nedd4 fluorescence intensity was measured along the blue and yellow dashed lines that cut through the region of the tectum where RGC axons arborize (see Experimental Procedures). Scale bar, 100 μm. (J) Graph showing Nedd4 pixel intensity along the dashed lines in (H). Yellow line represents Nedd4 signal in the tectum with no RGC axons, and the blue line represents Nedd4 signal in the tectum innervated by RGC axons.

First, we investigated if Nedd4 is expressed in RGC axons. To test for Nedd4 expression, we used an anti-Nedd4 antibody that was raised against the three WW domains of the mouse Nedd4-1 protein (Harvey et al., 1998). This antibody recognizes the full-length *Xenopus* Nedd4 protein (∼110 kD) as shown by western blotting (Figure 2D), as well as the smaller band (∼55 kD), which probably represents the proteolytically cleaved Nedd4 as previously reported (Harvey et al., 1998). Antibody specificity was further verified by its ability to detect a decrease in Nedd4 levels in antisense morpholino (Nedd4-MO) injected embryos (Figures 4A and 4B). Immunocytochemistry using this antibody on cultured retinal axons revealed that Nedd4 is abundantly present in axons and GCs (Figures 2A and 2B). Analysis of Nedd4 immunostaining in vivo showed that Nedd4 is expressed throughout the developing retina and is enriched in the dendritic inner and outer plexiform layers (IPL and OPL; Figure 2C). Importantly, Nedd4 is expressed in the optic fiber layer (OFL) and in the optic nerve head (ONH)—two regions that contain primarily RGC axons. In the brain, Nedd4 expression colocalizes with GFP-labeled RGC axons as they navigate to the tectum, strongly suggesting that Nedd4 is expressed in RGC axons at the time when they are arriving in the target area and branching (Figures 2E–2G).

To determine unequivocally that Nedd4 is present in RGC axons, as opposed to the cellular substrate on which the axons are growing, we compared the Nedd4 immunofluorescence signal intensity between the two tecta of the same brain, wherein one tectum was made devoid of RGC axons by unilateral eye removal early in development. The other tectum received normal innervation from the remaining (contralateral) retina, which had been electroporated with a GFP marker in order to make its RGC axons visible. As in the retina, Nedd4 expression is widespread in the brain (Figure 2H), and a comparison of Nedd4 immunofluorescence signal between the two tecta revealed a sharp decrease in Nedd4 signal in the tectum lacking RGC axons (Figures 2H–2J). As a further confirmation of Nedd4 expression, we performed in situ hybridization and found a distribution of Nedd4 mRNA similarly broad to that of Nedd4 protein (data not shown). A similarly widespread expression of Nedd4 has been previously reported in the developing mouse CNS (Kumar et al., 1997). Combined, the expression data presented here show that Nedd4 is widely expressed in the developing *Xenopus* retina and brain, including the RGC axons and GCs in vitro and in vivo at stages when these axons elaborate their terminal arbors, making Nedd4 a strong candidate component of axonal UPS for regulating branching in the tectum.

### Dominant-Negative Nedd4 Inhibits Axon Branching in the Tectum

In order to explore the functional role of Nedd4 in RGC axons, we sought to interfere with Nedd4 function. Nedd4 belongs to the HECT family of E3 ligases that contain an invariant Cys residue in their catalytic domain that forms a thiol-ester bond with the ubiquitin molecule before passing it on to a substrate. Mutations in this key catalytic Cys residue destroy all enzymatic function, and such mutant proteins act as dominant-negatives in yeast, fly, mouse, and human (Arévalo et al., 2006; Dunn and Hicke, 2001; Fotia et al., 2006; Myat et al., 2002). We therefore constructed a putative dominant-negative *Xenopus* Nedd4 (Nedd4-DN) by mutating the catalytic residue to Ala (C938A) and electroporated an N-terminal Myc-tagged Nedd4-DN cDNA directly into the developing retina to test if this affects branching of RGC axons. Embryos were then allowed to develop to stage 43/44, by which time the RGC axons have reached the tectum and begun to form arbors (Cohen-Cory and Fraser, 1995). As with UbK48R overexpression, expression of Nedd4-DN did not perturb long-range pathfinding; axons followed the normal optic pathway and reached the tectum correctly. However, the proportion of brains containing branched Nedd4-DN axons was greatly reduced compared with brains with control GFP axons, suggesting that Nedd4 has a role in retinal axon arborization (Figure S1, available online). Although the majority of Nedd4-DN axons reach the tectum approximately 10 hr later (by stage 41) than controls (by stage 40), this delay cannot account for the severe effect on branching seen at later stages 42 and 43. While control axons begin to arborize as soon as they have entered the tectum, Nedd4-DN axons remain unbranched for much longer periods of time (Figure S1).

**Figure 3:**
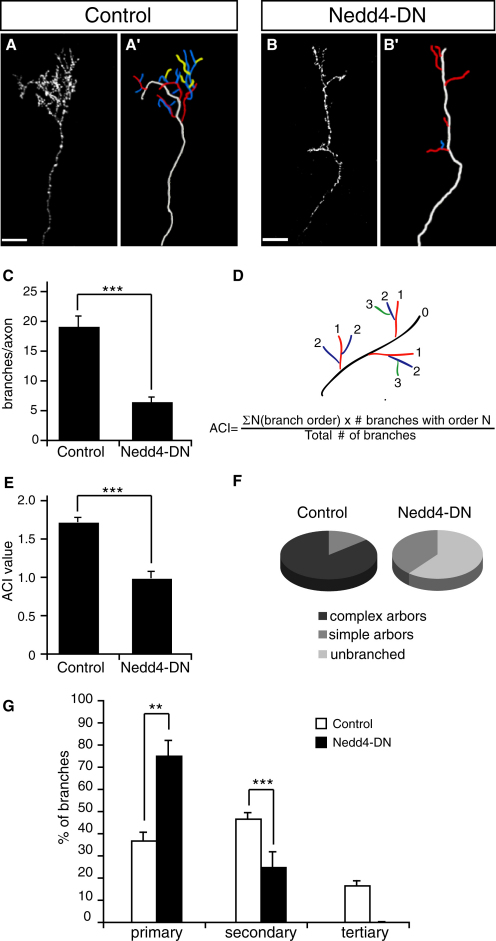
Nedd4-DN Inhibits Axon Branching in the Tectum (A and B) Lateral view of axons expressing control GFP (A) and Nedd4-DN (B) in the tectum. Corresponding axon trajectories are in (A′) and (B′), where branches of a different order are color coded: white, axon shaft; red, primary; blue, secondary; yellow, tertiary. Scale bars, 20 μm. (C) Graph showing the average number of branches per axon arbor. (D) Formula for Axonal Complexity Index, adapted from Marshak et al. (2007). (E) Graph showing the average ACI value per axon arbor. (F) Pie charts representing the proportions of unbranched axons and branched axons with simple and complex morphologies. Simple arbors, ACI < 1.4; complex arbors, ACI ≥ 1.4. (G) Graph showing the proportion of branches of different order in axon arbors. ^∗∗^p < 0.001, ^∗∗∗^p < 0.0001, Student's t test.

To gain a better understanding of how Nedd4 regulates branching, we analyzed the morphologies of individual Nedd4-DN-expressing arbors in the tectum. The difference in morphology between Nedd4-DN and control axon arbors was striking. While most control axons formed elaborate terminal arbors consisting of many branches, the majority of Nedd4-DN axons were unbranched, or contained very few branches (Figures 3A, 3B, and 3F).

We quantified this difference in several ways. First, analyzing total axon branch numbers revealed that Nedd4-DN axons had a significantly lower number of branches compared with control GFP axons (Figure 3C). Second, we quantified axon arbor complexity using the Axonal Complexity Index (ACI), which takes into account the number of branches of different order (Marshak et al., 2007) (Figure 3D). The average ACI value was significantly lower for Nedd4-DN axons compared with GFP controls (Figure 3E). Note that for control axons, both the overall number of branches and the average ACI value that we observe is similar to that previously reported for *Xenopus* RGC axons at a similar developmental stage (Marshak et al., 2007). Third, we classified arbors as unbranched, “simple,” or “complex” using the ACI value of 1.4 as a cutoff point between simple and complex. We chose this value because control axons with ACI < 1.4 did not contain any tertiary branches, which otherwise contribute the most to the increment of the ACI. The vast majority of control axons (85.7%) formed complex arbors, while the remaining 14.3% axons made simple arbors (Figure 3F). In contrast, the majority of Nedd4-DN expressing axons were unbranched (60.8%, p < 0.0001), and even those that branched formed simple arbors (39.1%, p < 0.05).

Finally, the low ACI of Nedd4-DN arbors suggests that they are composed mainly of primary branches. This is indeed the case, as can be seen from the branch order distribution analysis. While the control arbors are, for the most part, composed of secondary and tertiary branches, the contribution from secondary branches is significantly diminished in Nedd4-DN arbors, and the tertiary branches are even lacking altogether (Figure 3G). Taken together, these data suggest that interfering with Nedd4 activity in RGC axons inhibits their ability to branch in vivo.

### Nedd4 Morpholino Inhibits Axon Branching in the Tectum

**Figure 4:**
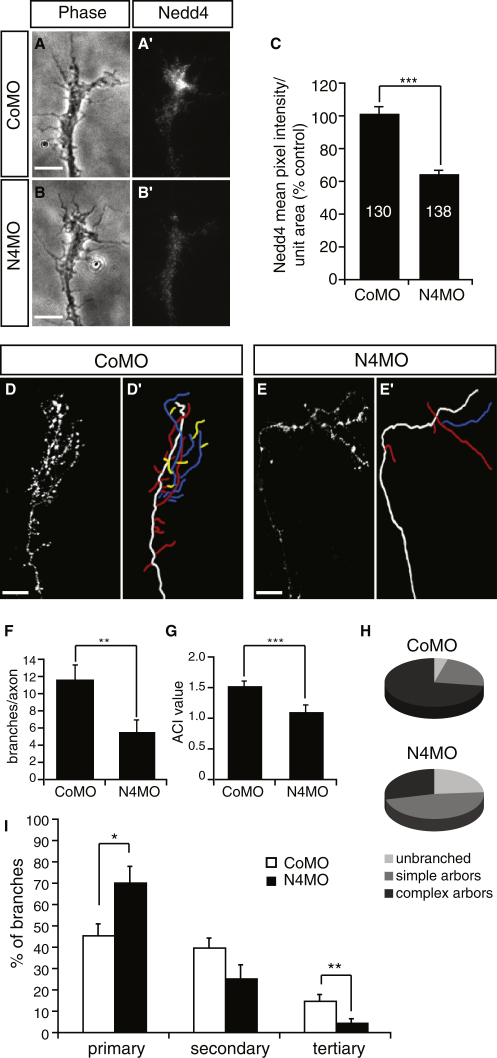
Nedd4-MO Inhibits Axon Branching in the Tectum (A–C) Nedd4-MO leads to a knockdown in Nedd4 protein levels in GCs. Nedd4 immunofluorescence in a representative GC positive for a Control-MO (A and A′) and Nedd4-MO (B and B′) is shown. Scale bars, 5 μm. (C) A graph showing the average Nedd4 signal intensity per unit area in Nedd4-MO versus Control-MO GCs. Numbers on the bars represent the number of GCs analyzed. ^∗∗∗^p < 0.0001, Student's t test. (D and E) Lateral view of Control-MO-positive (D) and Nedd4-MO-positive axons (E). Corresponding axon trajectories are in (D′) and (E′), where branches of a different order are color coded as before. Scale bars, 20 μm. (F) Graph showing the average number of branches per axon arbor. (G) Graph showing the average ACI value per axon arbor. (H) Pie charts representing proportions of Control-MO or Nedd4-MO axons with different morphologies. Simple arbors, ACI < 1.4; complex arbors, ACI ≥ 1.4. (I) Graph showing the proportion of branches of different order in axon arbors. ^∗^p < 0.01, ^∗∗^p < 0.001, ^∗∗∗^p < 0.0001, Student's t test.

We next used antisense morpholinos (MO) to downregulate Nedd4 and to further test its role in axon branching. First we established that the specific, FITC-tagged Nedd4-MO was effective in downregulating endogenous Nedd4 in RGC axons and GCs. MO blastomere injection has been shown to be an effective tool to knock down protein expression in retinal precursors and RGC GCs (Leung et al., 2006). Thus, we injected 10 ng of Nedd4-MO or control MO (Control-MO) into a dorsal blastomere at the 8-cell stage to introduce the MOs into the lineage that gives rise to half of the CNS on the injected side, including most of the retina (Moody, 2000). At this concentration, neither Nedd4-MO nor Control-MO had any adverse effects on embryonic development. Following injection, the MO (FITC)-positive retinae were explanted into culture at stage 35/36, and the Nedd4 fluorescence signal intensity was calculated quantitatively (mean pixel intensity/unit area) and compared between Nedd4-MO- and Control-MO-positive GCs 24 hr later. In this way the age of axons at the time of analysis corresponds to stage 40 in vivo (Campbell et al., 2001; Shewan et al., 2002), which is when most RGC axons reach the tectum and begin to arborize. Nedd4-MO led to a marked and specific downregulation in Nedd4 protein levels in the GC. The average Nedd4 signal intensity per unit area was 40% lower in Nedd4-MO-positive GCs compared with Control-MO-positive GCs (Figures 4A–4C). This effect was specific for Nedd4-MO, because the Control-MO did not significantly influence the levels of Nedd4 immunofluorescence in the GC (data not shown).

To study the effect of Nedd4-MO on axon branching, the MO-positive retinae (of MO-blastomere-injected embryos) were electroporated at stage 28 with a membrane-bound RFP cDNA in order to label individual RGC axons, and the embryos were allowed to develop to stage 43/44. However, we could not observe any obvious branching defects at this stage, possibly due to low effective MO concentration in RGCs at later developmental stages when branching occurs, and as a consequence of MO dilution with each cell division. We have previously shown that MOs can be successfully electroporated into the retina (Falk et al., 2007) and so we aimed to overcome the dilution effect by double introduction of the MO, first by blastomere injection, and second by coelectroporation into the retina at stage 28 along with the RFP. This sequential MO delivery is expected to increase the effective amount of MO in RGCs, and thus the knockdown of Nedd4, in axons branching in the tectum. The overall strategy of sequential MO delivery and RFP coelectroporation is outlined in Figure S2.

Quantification of colocalization of MO and RFP in retinal sections revealed that the majority of RFP cells also contained the MO (70% ± 1.7% for Nedd4-MO; 80% ± 2.6% for Control-MO) in accordance with the previous report (Falk et al., 2007). Thus, we could be confident that the majority of analyzed RFP-positive RGC arbors in the tectum also contain the MO.

When reapplied by electroporation, the Nedd4-MO significantly reduced the ability of axons to branch (Figures 4D and 4E). Both the overall number of branches (Figure 4F) and the average ACI (Figure 4G) were reduced in Nedd4-MO arbors compared with Control-MO arbors. Furthermore, we found that the majority of Nedd4-MO axons either formed simple arbors (47.6%, p < 0.05) or did not branch at all (23.8%, p < 0.05). This was in contrast to Control-MO axons, of which the vast majority developed complex arbors (72.7%), with only a small proportion forming simple arbors (22.7%) or remaining unbranched (4.5%) (Figure 4H). Notably, in the retina, 20%–30% of mRFP-positive cells did not express MO at detectable levels (data not shown). Of the 28.5% of Nedd4-MO arbors that had a complex, wild-type morphology, it is tempting to speculate that they were formed by RGCs lacking detectable levels of MO, and therefore possessing normal Nedd4 activity. Nedd4-MO arbors were less complex, because they mainly contained primary and secondary branches, with a significantly diminished contribution from tertiary branches (Figure 4I).

Together, our data show that downregulating Nedd4 by MO results in the same axon branching phenotype as that caused by overexpression of the dominant-negative mutant that blocks Nedd4 function. That is, axons with decreased Nedd4 activity branch less and develop arbors that have simple morphology with fewer higher-order branches.

### Nedd4 and PTEN Are Coexpressed in RGC Axons

**Figure 5:**
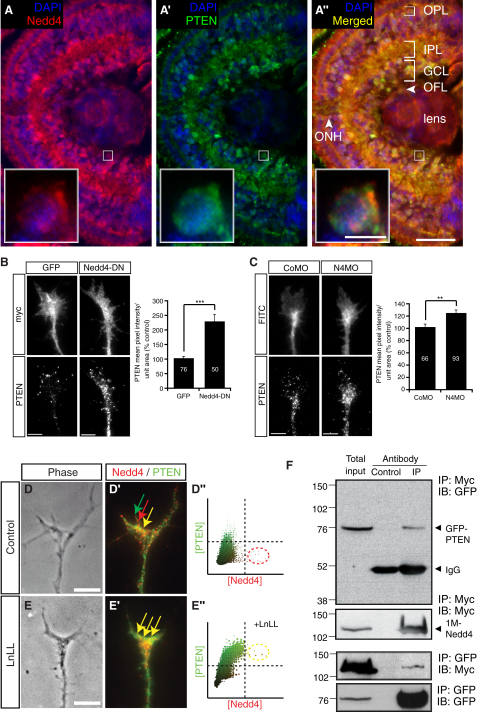
Nedd4 Regulates PTEN Levels in GCs (A) Transverse section through stage 40 retina stained with anti-Nedd4 (red) and anti-PTEN (green) antibodies. Blue signal indicates nuclear staining (DAPI). IPL, inner plexiform layer; OPL, outer plexiform layer; OFL, optic fiber layer; ONH, optic nerve head. Scale bars, 50 μm for (A″); 5 μm for (A″) inset. (B and C) Nedd4-DN (B) or Nedd4-MO (C) leads to an increase in PTEN signal intensity in GCs. (Left) Representative GCs containing the control constructs (Myc-GFP or Control-MO) or the experimental constructs (Myc-Nedd4-DN or Nedd4-MO) as shown in the upper panels, and the corresponding PTEN fluorescence in the lower panels. Scale bars, 5 μm. (Right) Graph showing average PTEN pixel intensity per unit. Numbers on the bars represent the number of GCs analyzed. ^∗∗^p < 0.001, ^∗∗∗^p < 0.0001, Student's t test. (D and E) Inhibiting proteasomes increases colocalization of Nedd4 and PTEN in GCs. Nedd4 (red) / PTEN (green) immunoflurescence (D′ and E′) and the resulting pixel-by-pixel intensity histogram (D″ and E″) in representative GCs (D and E) incubated for 16 hr without (D) or with (E) a proteasome inhibitor (50 μM LnLL) is shown. Scale bars, 5 μm. (F) Nedd4 and PTEN form complexes. *Xenopus* Nedd4 tagged with a Myc tag (1M-Nedd4) and *Xenopus* PTEN tagged with EGFP (GFP-PTEN) were transfected into HEK293T cells and their interactions were examined by coimmunoprecipitation using a control IgG (control) or an IP antibody (IP: anti-Myc for the upper two panels and anti-GFP for the lower two panels) followed by western blot (IB) using an anti-Myc or anti-GFP antibody.

We next asked what targets of Nedd4 might regulate axon branching. A recent report identified tumor suppressor PTEN as a substrate for Nedd4 in immortalized cells (Wang et al., 2007). PTEN is a negative regulator of the PI3K signaling pathway—a pathway well established in the regulation of neuronal cytoskeleton dynamics and axon turning and remodeling (Cosker and Eickholt, 2007). Furthermore, loss of PTEN in hippocampal and cerebellar neurons leads to excessive axon growth and ectopically branched dendrites, suggesting a negative role for PTEN in neurite growth and branching (Kwon et al., 2006). First we investigated if PTEN and Nedd4 are expressed by the same RGCs by performing immunohistochemistry using embryonic stage 40 retina sections. The specificity of the antibody in recognizing full-length *Xenopus* PTEN was verified by western blot (Figure 7A). Similarly to Nedd4, PTEN was expressed broadly in the retina and enriched in the OFL and OPL, which contain neuronal processes and synapses (Figure 5A). Importantly, Nedd4 and PTEN were coexpressed by the same RGCs (Figure 5A, insets) and are clearly abundant in the OFL and ONH; that is, in the regions that contain RGC axons as they navigate out of the eye (Figure 5A). Thus, PTEN and the E3 ligase Nedd4 are both expressed in the same RGC axons at the time when branching occurs.

### Nedd4 Regulates PTEN Levels in the GC

Given that PTEN is a known Nedd4 substrate (Wang et al., 2007), we next asked if PTEN levels in RGC axons are Nedd4 dependent. Because it is not technically feasible to use classical biochemical methods to demonstrate this directly in GCs due to the minute amounts of material, we used quantitative immunofluorescence (QIF) to measure endogenous GC PTEN in the context of diminished Nedd4 activity.

First, we injected Nedd4-DN RNA into blastomeres to overexpress Nedd4-DN in the retinal precursor lineage in order to obtain a large number of Nedd4-DN-expressing RGCs for analysis of their GCs in culture. The GCs were immunostained with both anti-Myc and anti-PTEN antibodies to detect the presence of Nedd4-DN and to visualize the PTEN fluorescence levels, respectively. Our results show that overexpression of Nedd4-DN dramatically increases PTEN signal intensity (over 2-fold), consistent with the previous findings that Nedd4 downregulates PTEN (Wang et al., 2007) (Figure 5B). We observe the same effect when Nedd4-MO is present in RGC axons (Figure 5C). The increase in PTEN is more modest in the presence of Nedd4-MO (∼20%) than in the presence of Nedd4-DN (200%), which probably reflects the different degrees to which Nedd4 function is disrupted. Indeed, we estimated that Nedd4-DN overexpression leads to a >10-fold increase in overall Nedd4 immunofluorescence (data not shown), whereas Nedd4-MO induces a comparatively modest change in Nedd4 levels, decreasing it by 40%. These data suggest that PTEN is downregulated by Nedd4 in the GC. Next we investigated how Nedd4 and PTEN are localized in GCs using cultured retinal axons. Although Nedd4 and PTEN were generally colocalized (Figure 5D′, yellow arrow), there were several regions in the GCs that contained high levels of Nedd4 (Figure 5D′, red arrow) or PTEN (Figure 5D′, green arrow) only. If Nedd4 is the E3 ligase for PTEN, their interactions will result in degradation of PTEN. Therefore we reasoned that colocalization of Nedd4 and PTEN will increase when the UPS is inhibited. Indeed, treatment with a proteasome inhibitor (25 μM N-Acetyl-Leu-Leu-NorLeu-Al [LnLL]) for 16 hr increased colocalization (Figure 5E′, yellow arrow) as represented in Nedd4-PTEN intensity histrograms (Figures 5D″ and 5E″) (Pearson's correlation coefficient = 0.62 ± 0.12 versus 0.93 ± 0.01; p = 0.0013, Student's t test).

Having established their colocalization in the RGC GCs, we performed a coimmunoprecipitation experiment using a heterologous expression system. We transfected *Xenopus* Nedd4 tagged with a Myc tag (1M-Nedd4) and *Xenopus* PTEN tagged with EGFP (GFP-PTEN) into HEK293T cells in which the interaction of mammalian Nedd4-1 and PTEN was first demonstrated (Wang et al., 2007). After incubation with proteasomal inhibitors, cells were lysed and Nedd4-PTEN protein complexes were immunoprecipitated and revealed by western blot using anti-Myc and GFP antibodies. Unlike the control IgG (Control), a Myc antibody (Figure 5F, upper two panels) efficiently precipitated 1M-Nedd4 (Figure 5F, second panel) and GFP-PTEN was coprecipitated by 1M-Nedd4 (Figure 5F, top panel). Likewise, immunoprecipitation of PTEN coprecipitated Nedd4 (Figure 5F, lower two panels), indicating that *Xenopus* Nedd4 and PTEN do form complexes when expressed in the same cells. This is in agreement with the published data showing that Nedd4 and PTEN physically interact (Ahn et al., 2008; Wang et al., 2007, 2008).

### Overexpression of PTEN Inhibits RGC Axon Branching

If Nedd4 downregulates PTEN in RGC axons in vivo, then overexpression of PTEN would be expected to phenocopy the effect of Nedd4-DN expression and Nedd4-MO on axon branching. To test this possibility we overexpressed GFP-PTEN in the developing retina by electroporation at stage 28, and allowed embryos to develop to stage 43/44, when we analyzed morphologies of individual GFP-PTEN-positive axon arbors in the tectum. Overexpression of GFP-PTEN has been shown previously to inhibit PI3K signaling in *Xenopus* embryos (Ueno et al., 2006).

**Figure 6:**
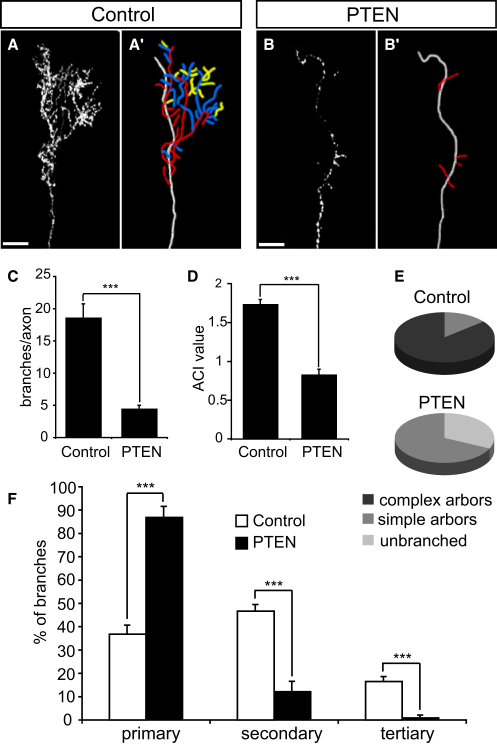
Overexpressed PTEN Inhibits RGC Axon Branching in the Tectum (A and B) Lateral view of control (GFP) (A) and GFP-PTEN (B) axons in the tectum with corresponding axon trajectories in (A′) and (B′). Axon trajectories are color-coded as before. Scale bars, 20 μm. (C) Graph showing the average number of branches per axon arbor. (D) Graph showing the average ACI value per axon arbor. (E) Pie charts representing proportions of axons with different morphologies. Simple arbors, ACI < 1.4; complex arbors, ACI ≥ 1.4. (F) Graph showing the proportion of branches of a different order in axon arbors. ^∗∗∗^p < 0.0001, Student's t test.

Axons expressing GFP-PTEN were severely affected in their ability to branch (Figures 6A and 6B). Both the total number of branches (Figure 6C) and the ACI (Figure 6D) were significantly reduced compared with those of control axons. As many as 32% of axons were unbranched (p < 0.005), whereas the remaining 68% formed simple arbors (p < 0.0005) (Figure 6E). There were no GFP-PTEN axons with complex arbors, in sharp contrast to GFP controls, in which 85.7% of axons formed complex arbors. Furthermore, GFP-PTEN arbors were mainly composed of primary branches. In fact, only 1 out of 26 analyzed GFP-PTEN axons had any tertiary branches at all (Figure 6F). Thus, our data show that overexpression of PTEN suppresses RGC axon branching, which is the same outcome that we observe following inhibition of Nedd4 activity. Given that Nedd4 was shown to ubiquitinate PTEN, and to target it for degradation in the proteasome (Wang et al., 2007), our data are consistent with a model wherein Nedd4 acts to promote branching by downregulating PTEN.

### Decreasing PTEN Levels Rescues the Branching Defect Caused by Nedd4 Inhibition

**Figure 7:**
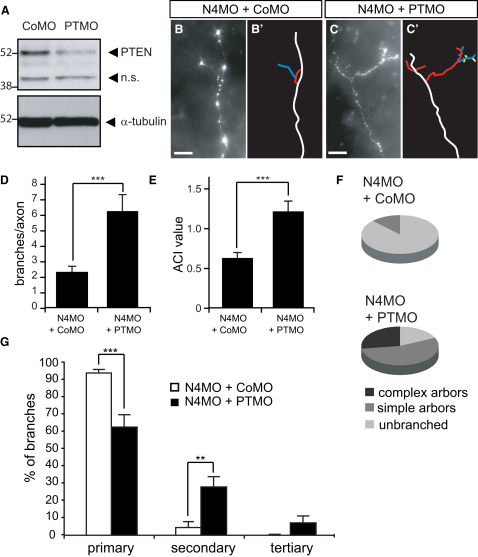
PTEN-MO Rescues the Branching Defect Caused by Nedd4-MO (A) PTEN-MO leads to a specific knockdown in PTEN protein levels in retina. Retinae isolated from embryonic stage 40 embryos injected with Control-MO or PTEN-MO were subjected to western blot using an anti-PTEN antibody (upper panel). (B–G) Nedd4-MO was cointroduced with Control-MO (B) or with PTEN-MO (C) to examine the branching phenotype. (B and C) Lateral view of Nedd4-MO+Control-MO-positive (B) and Nedd4-MO+PTEN-MO-positive axons (C). Corresponding axon trajectories are in (B′) and (C′), where branches of a different order are color coded as before. Scale bars, 20 μm. (D) Graph showing the average number of branches per axon arbor. (E) Graph showing the average ACI value per axon arbor. (F) Pie charts representing proportions of axons with different morphologies. Unbranched, ACI < 1; simple arbors, 1 ≤ ACI < 1.4; complex arbors, ACI ≥ 1.4 (G) Graph showing the proportion of branches of different orders in axon arbors. ^∗∗^p < 0.001; ^∗∗∗^p < 0.0001, Student's t test.

If loss of Nedd4 function leads to increased PTEN levels and consequent branching defect, then reducing PTEN should rescue the Nedd4 branching phenotype. In order to test this possibility, we used antisense MOs to downregulate PTEN and Nedd4 simultaneously. First, the specificity of PTEN-MO tagged with FITC (PTEN-MO) was confirmed after blastomere injections as described for Nedd4-MO (Figures 4A–4C). Following injection, the MO (FITC)-positive retinae were dissected and PTEN levels were examined by western blot analyses. PTEN-MO led to a marked and specific downregulation in PTEN protein levels (Figure 7A: compare the PTEN bands with the nonspecific bands or with the α-tubulin bands). Next, we examined if PTEN-MO reduces the effect of Nedd4-MO on axon branching using sequential MO delivery as described before (Figure 4 and S2) using Nedd4-MO+Control-MO (Nedd4-only inhibition) or Nedd4-MO+PTEN-MO (Nedd4 and PTEN dual inhibition). Indeed, PTEN-MO, but not Control-MO, significantly increased the ability of Nedd4-MO-containing axons to branch (Figures 7B and 7C). Both the overall number of branches (Figure 7D) and the average ACI (Figure 7E) were increased in Nedd4-MO+PTEN-MO arbors compared with Nedd4-MO+Control-MO arbors. Moreover, we found that many Nedd4-MO+PTEN-MO axons formed complex arbors (27.3%), whereas no complex arbors were observed in Nedd4-MO+Control-MO axons (Figure 7F). The more severe branching defect by Nedd4-MO alone in this experiment compared with Figure 4 is likely due to different amount of Nedd4-MO (10 ng for Figure 4 and 20 ng for Figure 7). Nedd4-MO+PTEN-MO arbors became more complex because the contribution of secondary and tertiary branches increased (Figure 7G).

We further investigated functional interactions between Nedd4 and PTEN in axon branching by coelectroporating a wild-type PTEN tagged with GFP (PTWT) or a dominant-negative PTEN tagged with GFP (PTDN). Our model predicts that further increasing PTEN levels in Nedd4-DN-expressing axons will enhance the branching defect, and inhibiting PTEN function will reduce the branching defect. In order to test this possibility, we expressed PTWT, PTDN, or GFP in Nedd4-DN-expressing RGC axons in developing retina by electroporating at stage 28 and allowing embryos to develop to stage 43/44, at which point we analyzed the branching pattern as described above.

In accordance with our model, axons expressing PTWT with Nedd4-DN had a branching defect much more severe than that of axons expressing Nedd4-DN alone (Figures S3A and S3B). In contrast, axons coexpressing PTDN slightly recovered their ability to branch (Figure S3C). Both the total number of branches (Figure S3D) and the average ACI (Figure S3E) reflected the functional interactions between Nedd4 and PTEN in regulating axon branching. Complex arbors were observed only in Nedd4-DN+PTDN axons (33%) (Figure S3F), which resulted from increased contribution of secondary and tertiary branches in these axons (Figure S3G). Therefore, our data strongly support the model in which Nedd4 promotes axon branching by targeting PTEN to proteasomal degradation.

### Netrin-1 Activates UPS-Dependent Degradation of PTEN

**Figure 8:**
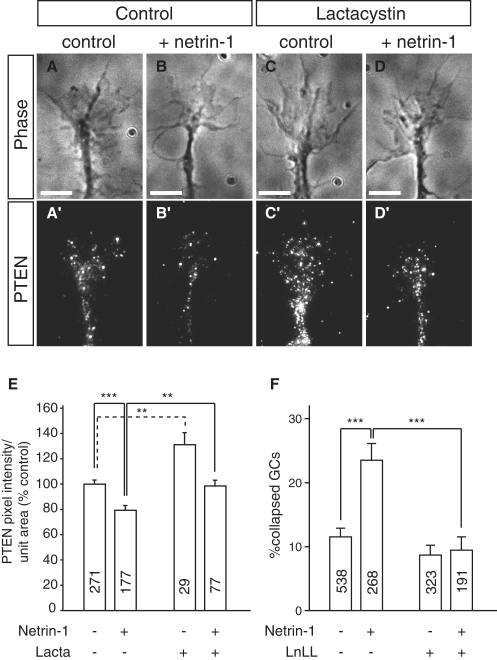
Netrin-1 Induces UPS-Dependent PTEN Degradation and GC Collapse Stage 35/36 retinal explants were cultured for 24 hr, after which they were stimulated for 5 min with 3 μg/ml recombinant human Netrin-1, after which they were fixed and stained with an anti-PTEN antibody. (A–D) Images of representative GCs unstimulated (A and C) or stimulated with Netrin-1 (B and D). Lactacystin (10 μM) was added to cultures immediately prior to stimulation in (C) and (D). Scale bars, 5 μm. (E) Graph showing quantification of average PTEN pixel intensity per unit area normalized to the unstimulated control. The numbers in the bars represent numbers of analyzed GCs. ^∗∗^p < 0.001; ^∗∗∗^p < 0.0001, Student's t test. (F) Stage 32 retinal explants were cultured for 24 hr on a high laminin (20 μg/ml) substrate. Axons were stimulated with 1.2 μg/ml Netrin-1, or control buffer in which Netrin-1 was dissolved. After 10 min they were fixed and the number of collapsed GCs was counted. The numbers in the bars represent numbers of analyzed GCs. ^∗∗∗^p < 0.0001, Dunn's Multiple Comparisons Test followed by Kruskal-Wallis test.

Terminal branching in retinal axons is regulated in vivo by extrinsic factors such as Netrin-1, BDNF, and Slits (Campbell et al., 2007; Manitt et al., 2009; Marshak et al., 2007). Therefore, we sought to determine whether at least one such a factor could link UPS function to branching. Because Netrin-1 induces UPS-dependent chemotropic turning responses in RGC GCs in vitro (Campbell and Holt, 2001), we investigated whether Netrin-1 triggers changes in PTEN levels. We tested this using QIF on GCs in vitro and found that Netrin-1 stimulation for 5 min induced a robust ∼30% decrease in the levels of PTEN signal, indicating that PTEN is rapidly degraded (Figures 8A–8E). Indeed, the Netrin-1-induced decrease in PTEN was blocked by proteasomal inhibitor treatment (Figure 8E). Inhibitor treatment alone caused a significant increase in PTEN consistent with our results showing Nedd4-mediated PTEN degradation.

To investigate whether there is a functional link between Netrin-1 and the UPS, we used the GC collapse assay, as there is no robust branching assay available for these axons and several studies indicate a link between the collapse of GCs and the branching (Brose and Tessier-Lavigne, 2000; Campbell et al., 2001; Wang et al., 1999). When applied to RGC GCs under conditions of high laminin, Netrin-1 causes significant collapse within 10 min (Campbell and Holt, 2003; Piper et al., 2005; Shewan et al., 2002). We tested whether this response required the UPS and found that, indeed, collapse was abolished with proteasomal inhibitor treatment (Figure 8F).

These results indicate that signaling downstream of Netrin-1 includes rapid PTEN degradation. Moreover, Netrin-1-induced GC collapse is UPS-dependent, suggesting that Netrin-1 in the tectum may induce Nedd4-dependent PTEN degradation to activate a signaling cascade that leads to increased axon branching.

## Discussion

**Figure 9:**
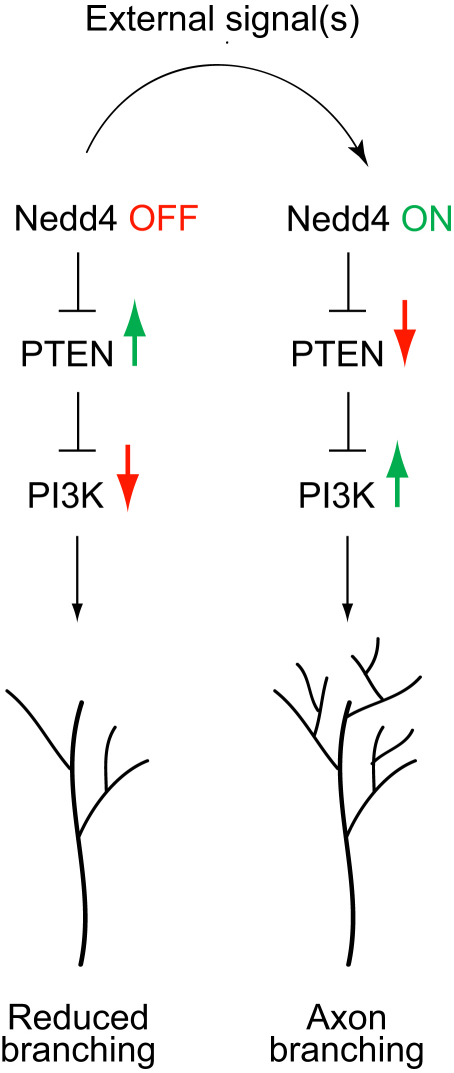
A Proposed Model for How Nedd4-Mediated Downregulation of PTEN Promotes PI3K Signaling in an Axon and Its Branching In this model, external signals promote or reduce axon branching by modulating Nedd4-dependent PTEN downregulation, and consequently, the levels of downstream PI3K signals. An increase in Nedd4 activity results in reduced PTEN levels and therefore promotes the PI3K pathway and the downstream cytoskeletal rearrangements that favor branch growth. Conversely, an elevation in PTEN levels due to compromised Nedd4 activity inhibits PI3K signaling and reduces arbor growth.

Previous studies showed that guidance cues elicit local activation of the UPS in RGC GCs and that UPS activity is required for Netrin-1 gradient-induced turning in vitro (Campbell and Holt, 2001). Here we have addressed the in vivo role of the UPS in RGC axon development, and our functional studies show that UPS function is not required for long-range pathfinding but is essential for terminal branching in the tectum. We identify Nedd4 as an E3 ligase that regulates axon branching and we show that it associates with and degrades PTEN, a known substrate. We demonstrate that PTEN is epistatic to Nedd4 in axons because downregulation of PTEN rescues the Nedd4-loss-of-function branching defect, and PTEN upregulation augments the phenotype. Finally, Netrin-1 triggers a rapid UPS-dependent decrease in PTEN in GCs, suggesting a model in which extrinsic cues control branching by activating Nedd4, which downregulates PTEN and permits/stimulates branching, whereas Nedd4 inactivation leads to PTEN accumulation and disables branching (Figure 9).

The data presented here suggest that the UPS and Nedd4 have a specific role in regulating RGC axon branching and not their long-range guidance through the optic pathway to the tectum. Axons with perturbed UPS or Nedd4 activity made correct decisions at multiple pathway choice points, including the correct exit out of the retina at the ONH, appropriate crossing at the optic chiasm, normal trajectories in the optic tract, and entry into the tectum. In contrast, RGC axons require UPS activity for proper in vitro directional turning responses to guidance cues such as Netrin-1, which is expressed at multiple points in the pathway, including the ONH and tectum (de la Torre et al., 1997), and BDNF, which is expressed in the tectum (Cohen-Cory and Fraser, 1994). This apparent discrepancy could potentially be reconciled if the in vitro turning responses actually reflect the mechanism that underlies GC behavior in the tectum, where the short-range selection of synaptic partners occurs, rather than that which underlies the long-range process of pathfinding. Support for this idea comes from studies on BDNF. BDNF promotes branching of RGCs in vivo (Alsina et al., 2001; Cohen-Cory and Fraser, 1995) whereas axons turn toward the source of BDNF when applied as a gradient in vitro (Campbell and Holt, 2001). Thus BDNF at least can act as both an attractive guidance cue and as a promoter of branching. While this might suggest that BDNF could be required for long-range axon guidance, in vivo RGC axons do not encounter BDNF until they reach the tectum (Cohen-Cory and Fraser, 1994). Furthermore, RGCs expressing the dominant-negative TrkB receptor pathfind normally from the retina to the tectum, but they fail to branch once they are in the target area (Marshak et al., 2007), consistent with the specific role of BDNF in regulating axon branching. When the data are taken together, then, there is clear precedent for a guidance cue that promotes branching and acts as a short-range, rather than long-range, attractant. Thus, by the same token, it appears that the UPS- and Nedd4-mediated types of degradation of PTEN are specifically required for branching in the tectum, and do not have any effect on the long-range guidance.

Axon branching is essential for the proper development and function of neuronal circuits because branches are, by and large, the contact sites between different neurons, and are thus the sites at which synapses are made. Furthermore, axon branching and synaptogenesis occur concomitantly as branches are preferentially sprouted from stable synaptic sites (Meyer and Smith, 2006; Ruthazer et al., 2006). The UPS has a well established role in axon remodeling during synaptogenesis, and a number of E3 ligases have been identified with a role in this process (Yi and Ehlers, 2007), including the members of the RPM-1/HIW/PHR-1 family of E3 ligases and anaphase promoting complex/cyclosome (APC/c), a multimeric E3 ligase complex, which attenuate branching and synaptogenesis in worms, flies, and mammals (Bloom et al., 2007; DiAntonio et al., 2001; Konishi et al., 2004; Lewcock et al., 2007; Schaefer et al., 2000; van Roessel et al., 2004; Wan et al., 2000; Zhen et al., 2000). In contrast to these E3 ligases, Nedd4 seems to promote, rather than attenuate, axon branching in a cell-autonomous manner. Interestingly, a recent study by Liu and colleagues demonstrated a non-cell-autonomous role for Nedd4 in the development of the mammalian NMJ (Liu et al., 2009). Although the molecular mechanism is likely to be different from the retinotectal synapse because only skeletal muscles and not motor neurons expressed Nedd4, it raises an interesting possibility that postsynaptic tectal neurons may express Nedd4 target molecules, which control presynaptic terminal maturation.

Our finding that Nedd4-PTEN interactions occur in GCs is consistent with previous reports that show a physical interaction between Nedd4 and PTEN (Ahn et al., 2008; Wang et al., 2007). In a recent paper, however, Fouladkou and colleagues reported that neither PTEN protein levels nor PTEN subcellular localization change in *Nedd4-1* knockout mouse embryonic fibroblasts (MEFs) (Fouladkou et al., 2008). Furthermore, they could not detect biochemical interactions between Nedd4-1 and PTEN. It was suggested by the authors that this might be due to the lack of the PY motif in PTEN, through which many known substrates bind to the WW domain of the Nedd4 family E3 ligases, including Nedd4-1. In contrast, Wang et al. reported that Nedd4-1 and PTEN binding does not occur through the WW domain-PY motif interactions (Wang et al., 2008). Similarly, other Nedd4 family E3 ligases, such as Rsp5 (Lee et al., 2009) and Smurf2 (Narimatsu et al., 2009), do bind to substrates that do not possess a PY motif, including PTEN (Narimatsu et al., 2009). Therefore one may speculate that other E3 ligases may compensate for the loss of Nedd4-1 in some cell types (e.g., in MEFs as in Fouladkou et al., 2008) but not in others (e.g., in HEK293T and in the prostate cancer cell line DU-145, as in Wang et al., 2007), depending on the expression profile of Nedd4 family E3 ligases. Indeed, strong evidence showing that Nedd4 family E3 ligases do interact with PTEN has been provided in a recent study showing biochemical and functional interactions between Nedd4-1 and PTEN (Yim et al., 2009). Our data clearly demonstrate that *Xenopus* Nedd4 and PTEN are colocalized in the RGC GCs and that they form biochemical complexes when coexpressed. Moreover, Nedd4 negatively regulates PTEN levels. Thus, although mammalian Nedd4-1 was originally identified as the E3 ligase for PTEN (Wang et al., 2007), our data strongly support the idea that *Xenopus* Nedd4 (more similar to Nedd4-2) regulates PTEN stability. Nedd4 seems to be the major E3 ligase involved in RGC axon branching because the severity of the branching defect increased with higher doses of Nedd4-MO (10 ng for Figure 4E and 20 ng for Figure 7B), indicating that Nedd4 function is not compensated for by other E3 ligases in RGC axons. Our results do not exclude the possibility that Nedd4 regulates other substrates in addition to PTEN.

How could Nedd4-mediated PTEN degradation affect cytoskeletal dynamics? PTEN dephosphorylates PIP3 to PIP2 and therefore decreases the amount of second messenger PIP3 and attenuates PI3K signaling (Maehama and Dixon, 1998). Downstream effectors of the PI3K pathway include the well established regulators of the cytoskeleton, such as the members of the RhoA small GTPase family (Cosker and Eickholt, 2007) and glycogen synthase kinase 3 beta (GSK3β) (Crowder and Freeman, 2000), and the activation of the PI3K pathway is generally associated with neurite outgrowth, whereas its inhibition generally leads to neurite retraction and cessation of growth. Thus, PTEN activity inversely correlates with the ability of neurons to sprout neurites, and it is likely to be tightly regulated during development. Intriguingly, PTEN has also been found to modulate branching morphogenesis in other systems, such as the kidney (Kim and Dressler, 2007).

Many different cues and receptors regulate RGC axon branching, including Netrin-1 (Manitt et al., 2009), BDNF (Alsina et al., 2001; Cohen-Cory and Fraser, 1995), EphrinA (Marler et al., 2008; Roskies and O'Leary, 1994), Sema3A (Campbell et al., 2001), and Slit1a (Campbell et al., 2007). Netrin-1 is a particularly interesting candidate with a potential role upstream of Nedd4 because its signaling requires the UPS (Campbell and Holt, 2001). Indeed, our data show that Netrin-1 induces proteasomal degradation of PTEN. The changes in PTEN signal correlate functionally with Netrin-1-induced GC collapse (which we used as a readout of the axon tip's responses to extrinsic cues): GCs with low PTEN collapse (and then may branch), whereas GCs in which proteasomes are inhibited cannot degrade PTEN and lose their ability to respond to Netrin-1. Interestingly, Unc-6/Netrin also induces axon branching and synaptogenesis by acting on Unc-40/DCC in *C. elegans* (Colón-Ramos et al., 2007; Gitai et al., 2003), suggesting that Netrin/DCC signaling may have a conserved role as a branching/synaptogenesis factor. Nedd4 family E3 ligases contain a Ca^2+^ binding C2 domain. Ca^2+^ influx, which results in the binding of Ca^2+^ to the C2 domain, activates the relocalization process of Nedd4 to the plasma membrane (Plant et al., 1997) where PTEN, its substrate, localizes to function. Because the activation of DCC by Netrin-1 induces opening of voltage-gated Ca^2+^ channels and TRPC1 channels (Wang and Poo, 2005), local activation of DCC at the axon terminal may induce Ca^2+^-dependent PTEN degradation by Nedd4. Therefore, we propose that Netrin-1 expressed and released in the tectum may play a role as a branching/synaptogenesis factor to the RGC axons by activating the Ca^2+^-Nedd4-PTEN/PI3K pathway.

In addition to activating the UPS, guidance cues can modulate protein levels by promoting local protein synthesis. Whereas Netrin-1 signals through both protein synthesis and degradation (Campbell and Holt, 2001), Slit2 (Piper et al., 2006) and Sema3A (Campbell et al., 2001) signaling do not depend on the UPS and instead require intact protein synthesis pathways, including the mammalian target of rapamycin (mTOR) pathway, which is activated by PI3K signals and regulates cap-dependent protein translation initiation (Guertin and Sabatini, 2007; Hay, 2005). In this sense PTEN is a key negative regulator of protein synthesis (via mTOR), which raises the intriguing possibility that an interplay between protein synthesis and degradation modulates the levels of signaling components in the dynamic process of axon branching. Local protein synthesis via mTOR and local UPS-mediated degradation are known to play roles in the chemotropic responses of *Xenopus* GCs (Campbell and Holt, 2001; Leung et al., 2006; Yao et al., 2006), yet it is not clear how these two processes are coordinately regulated. Given that the PI3K-mTOR pathway was shown to regulate branching of hippocampal dendrites (Jaworski et al., 2005) and that PTEN deletion in adult RGCs promotes axon regeneration in an mTOR-dependent manner following optic nerve injury (Park et al., 2008), our work suggests that a similar mechanism may also shape the morphology of presynaptic terminals. Nedd4 may therefore promote branch growth by degrading PTEN and allowing translation of mRNAs encoding growth related proteins, such as cytoskeletal proteins and regulators. Netrin-1 elicits local protein synthesis in axons (Campbell and Holt, 2001) and it is possible that part of its ability to induce branching is via the mTOR pathway. In future studies it will be interesting to investigate whether Nedd4-PTEN control of axon branching requires protein synthesis and how this might be regulated by different upstream signals.

## Experimental Procedures

### Embryos

*Xenopus* embryos were obtained by in vitro fertilization, raised in 0.1X Modified Barth's Saline at 14°C–20°C, and staged according to the tables of Nieuwkoop and Faber (1967).

### DNA Constructs and Morpholinos

All constructs used in this study were expressed in the pCS2+ vector (David Turner, University of Michigan, Ann Arbor). Membrane GFP and RFP were previously described (Das et al., 2003; Poggi et al., 2005). Human ubiquitins (UbK48R and UbWT, 99% identical to the *Xenopus* protein) were obtained from Dr. Ron Kopito and subcloned with an N-terminal Myc tag. *Xenopus* full-length Nedd4 cDNA (IMAGE clone No. 7008311, Accession number BC074133.1) was purchased from GeneService and subcloned with an N-terminal Myc tag. Nedd4-DN was constructed using the site-directed mutagenesis kit (Strategene) with the following primers: 5′-GCCCAGAGCTCACACATGCTTTAACCGACTGGACTTACC-3′ and 5′-GGTAAGTCCAGTCGGTTAAAGGCTGTGTGAGCTCTGGGC-3′ that substituted Cys-938 into alanine. RT-PCR was used to clone the full-length *Xenopus* PTEN from stage 17 embryo cDNA library. The full-length *Xenopus* PTEN (Accession number AF144732.1) was then tagged N-terminally with GFP and subcloned into pCS2+ as described previously (GFP-PTEN) (Ueno et al., 2006). The dominant-negative PTEN was constructed by deleting the phosphatase domain from the full-length *Xenopus* PTEN, and then subcloned into pCS2+ with an N-terminal GFP tag using the following primers: 5′-AAGAATTCGAATACAGACCGGTGCCC-3′ and 5′-AAGGATCCTCAGACTTTTGTAATTTGT-GTGA-3′. Antisense Nedd4-MO, PTEN-MO, and Control-MO conjugated to FITC were designed and supplied by GeneTools (Philomath, OR): *Xenopus* Nedd4-MO, 5′-TACCGCCGACTTGGGTAGATACCTG-3′; *Xenopus* PTEN-MO, 5′-CGAACTCCTTGATGATGGCGGTCAT-3′; Control-MO, 5′-CCTCTTACCTCAGTTACAATTTATA-3′.

### Embryo Injection and Electroporation

Embryos were injected as previously described (Vignali et al., 2000). Injections were performed at the 8-cell stage in one or both dorsal-animal blastomeres. Capped mRNAs were synthesized from linearized plasmids using mMESSAGE mMACHINE kit (Ambion) and 125 pg of Myc-Nedd4-DN mRNA or control Myc-GFP mRNA was injected. Nedd4-MO and Control-MO were injected at 10 ng each for Figure 4. For the rescue experiments (Figures 7 and S3), Nedd4-MO, PTEN-MO, and Control-MO were injected 20 ng each.

Embryos were electroporated as previously described (Falk et al., 2007). For scatter-labeling, 5 nl of 1 μg/μl DNA in water was injected into a stage 28 retinal primordium, followed by a single electric pulse (25 ms long) delivered at 16 V. For DNA and MO coelectroporation, DNA and MO were diluted in water to reach a final concentration of 1 μg/μl DNA (RFP) and 1 mM MO, and 5 nl of the solution was injected into the retina, followed by a single electric pulse (50 ms long) delivered at 18 V. Because cDNA constructs are expressed within 6 hr of transfection (Holt et al., 1990), constructs electroporated into stage 28 retinal primordia were expressed in axons pathfinding to the tectum (Figure S1 and data not shown).

### Retinal Cultures and Netrin-1 Stimulation

Eye primordia were dissected and cultured at 20°C for 24 hr in culture medium (60% L15 + antibiotics, GIBCO) on glass coverslips coated with poly-L-lysine (10 μg/ml, Sigma) and laminin (10–20 μg/ml, Sigma). For PTEN quantification, human Netrin-1 (3 μg/ml, Axxora) was used to stimulate stage 35/36 retinal explants (cultured for 24 hr) for 5 min before fixing and immunostaining for PTEN. Lactacystin (10 μM, Calbiochem) was applied immediately prior to Netrin-1 stimulation to inhibit proteasomal activity. For collapse assays, retinae were isolated from stage 32 embryos and cultured for 30 hr. Human recombinant Netrin-1 (1.2 μg/ml, R&D Systems) or control culture medium was added, and after 10 min the cultures were fixed. The number of collapsed GCs was counted and values were presented as percentage of GC collapse ± SEM. GCs with one or no fillopodia were regarded as collapsed GCs.

### Immunofluorescence

Twenty-four hour cultures of stage 35/36 retinal explants positive for Myc-Nedd4-DN, Myc-GFP, or Nedd4-MO were subjected to quantitative immunofluorescence as described elsewhere (Piper et al., 2006) using the following primary antibodies: mouse anti-Myc clone 9E10, 1:2000, Sigma; rabbit anti-Nedd4, 1:2000, BD Biosciences; rabbit anti-Nedd4-2, 1:400, Abcam; rabbit anti-PTEN, 1:10, SantaCruz; and goat anti-PTEN, 1:200, IMGENEX. Nedd4-PTEN colocalization analyses in GCs were performed using the Color Inspector 3D plugin of ImageJ (National Institute of Health). Briefly, Regions of Interest (ROIs) were selected from thresholded Nedd4 images, and Nedd4-PTEN intensities in each pixel within ROIs were represented in a 2D histogram using Wu quantification. Pearson's correlation coefficient between Nedd4 and PTEN levels was calculated using Excel (Microsoft). Immunohistochemistry of retinal sections and whole-mount brains was performed as described previously (Walz et al., 1997). For Nedd4 fluorescence line profiling, whole-mount stage 40 brains immunostained for Nedd4 were split along the ventral midline and flat mounted. A straight line was drawn to connect the two most posterior points in the dorsal and ventral half in each tectum. A new line was drawn, at a 45° angle to the previous line, so that it cut through each tectum at a level where RGC axons arborize. The length of both lines was the same. Nedd4 pixel intensity profiling was done along the latter lines in Openlab (Improvision), and the data were analyzed in Excel (Microsoft). Antibodies were applied at dilutions described above.

### Western Blot Analysis

Stage 40 embryos' heads were lysed in RIPA buffer (Sigma), resolved by 10% SDS-PAGE, transferred to a nitrocellulose membrane (BioRad), and subjected to western blot analyses using a rabbit anti-Nedd4 (1:2000, BD Biosciences) or goat anti-PTEN (1:1000, IMGENEX) antibody followed by an HRP-conjugated secondary antibody (Zymed) incubation and ECL-based detection (GE Healthcare).

### Coimmunoprecipitation

We used a heterologous expression system to examine biochemical interactions between *Xenopus* Nedd4 and PTEN. HEK293T cells, which were maintained in DMEM supplemented with 10% fetal bovine serum and 1% penicillin-streptomycin, were transfected with Myc-tagged Nedd4 and GFP-tagged PTEN expression vectors using Lipofectamine 2000 (Invitrogen) according to the manufacturer's instructions. After 24 hr transfected cells were treated with 25 μM LnLL for 16 hr before they were lysed in IP buffer (50 mM HEPES [pH 7.4], 50 mM NaCl, 1 mM EDTA, 1 mM EGTA, and 1% Triton X-100) supplemented with protease inhibitor cocktail (Sigma) and 50 μM LnLL. Lysate (1 mg/ml) was precleared with 40 μl Protein G-Sepharose (Sigma) for 1 hr at 4°C with gentle rotation. Precleared lysate was incubated with the appropriate primary antibody (mouse anti-Myc clone 9E10, 2 μg/ml, Sigma; anti-GFP mouse monoclonal antibody, 2 μg/ml, Roche; or purified control mouse IgG, 2 μg/ml, Invitrogen). After 16 hr of incubation, 40 μl of Protein G-Sepharose was added and the samples were incubated for 1 hr. After washing three times with IP buffer, protein-antibody complexes were subjected to western blot analyses using anti-GFP (1:500, Roche) and anti-Myc (1:3000, Sigma) antibodies as described above.

### Analysis of Axon Branching

Axon arbors in the tectum expressing fluorescently labeled proteins were excited with 488 nm and 561 nm lasers and imaged by the Leica SP2 confocal microscope using 20x or 63x water immersion objectives. 3D stacks of retinal arbors were collected at 1 μm (for 20x images) or 0.4 μm thick (for 63x images) optical sections, with each image representing the average of three scans, using Leica Confocal Software. For crude analysis of axon branching, 20x images of brains with axons in the tectum were analyzed for the presence of branched axons. Only brains that had one or more branched axons in the tectum were scored as “branched,” while brains with only unbranched axons were scored as “unbranched.” For analysis of axon arbor morphology, 3D projections of single axon arbors acquired at 63X or 40X were reconstructed using Volocity (Improvision) or by manually tracing each image along the z axis using OpenLab (Improvision). Arbors were traced manually and the number of branches was counted, taking note of the order of each branch. Only neurites whose length was 5 μm or more were considered as branches. The complexity of an arbor was calculated using the ACI (Marshak et al., 2007). At least 20 individual axons were analyzed for each sample set. All results are presented as mean ± SEM.
