# Supplementary material for: E3 Ligase Nedd4 Promotes Axon Branching by Downregulating PTEN
Source: Neuron. 2010 Feb 11;65(3):341–57. doi: 10.1016/j.neuron.2010.01.017 (PMC2862300; doi:10.1016/j.neuron.2010.01.017)
Supplement: Document S1. Supplemental Figures [file mmc1.pdf]

Neuron, volume *65*

Supplemental Information

E3 Ligase Nedd4 Promotes Axon Branching by Downregulating PTEN

Jovana Drinjakovic, Hosung Jung, Douglas S. Campbell, Laure Strochlic, Asha Dwivedy, and Christine E. Holt

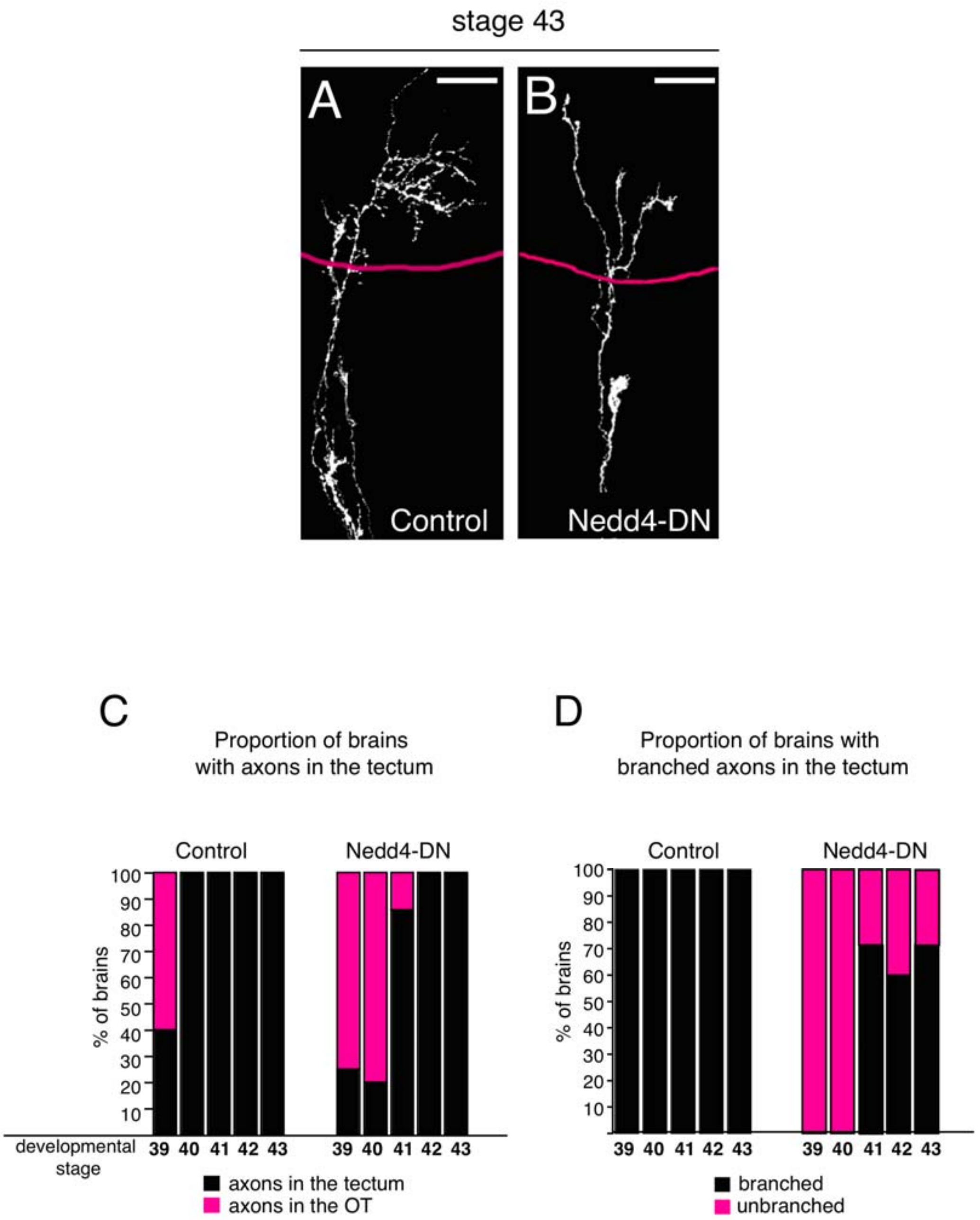

Supplementary Figure 1 **Nedd4-DN inhibits axon branching in the tectum**

(A-B) Lateral view of stage 43 brains containing axons expressing control GFP (A) and Nedd4-DN (B). The line demarcates the approximate anterior tectal boundary. Scale bars, 20 μm. (C) Graph showing the proportion of brains with axons expressing a given construct at different developmental stages. (D) Graph showing the proportion of brains with branched axons expressing a given construct at different developmental stages. At least 20 brains were analysed for each construct and stage.

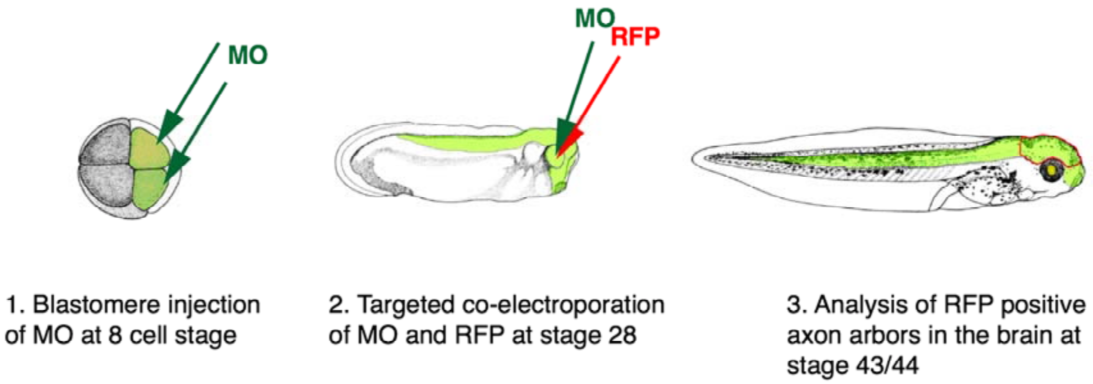

Supplementary Figure 2 **Sequential morpholino delivery**

Sequential MO delivery comprises two steps during which MO is administered during development in order to overcome the dilution effect caused by cell division and to increase the effective Nedd4 knock-down at later stages when branching occurs. First, MO is injected into two blastomeres at 8 cell-stage to introduce the MO to the CNS, including the retinae (1). Then, at stage 28 MO is co-electroporated, along with RFP marker to label individual axons, directly into the retina (2). Embryos are then fixed at stage 43/44, when RFP-positive axon arbors are analysed for their morphology (3).

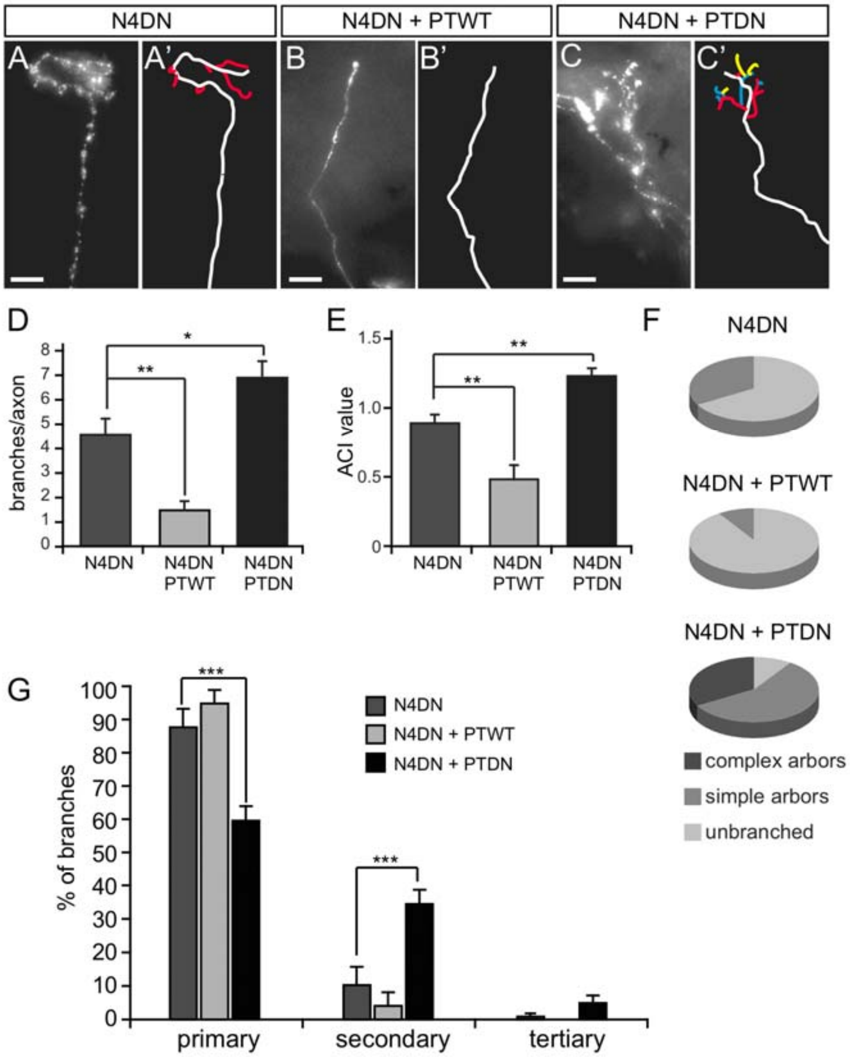

Supplementary Figure 3 Functional interactions between Nedd4 and PTEN in regulating axon branching

(A-B) Lateral view of axons co-expressing Nedd4-DN (N4DN) with GFP (A), with wildtype PTEN tagged with GFP (PTWT) (B), or with dominant negative PTEN tagged with GFP (PTDN) in the tectum. Corresponding axon trajectories are in A', B', and C' where branches of a different order are colour coded as before. Scale bars, 20 μm. (D) Graph showing the average number of branches per axon arbor. (E) Graph showing the average ACI value per axon arbor. (F) Pie charts representing proportions of axons with different morphologies. Unbranched, ACI<1; Simple arbors, 1<ACI<1.4; complex arbors, ACI>1.4. (G) Graph showing the proportion of branches of different order in axon arbors. ***p<0.0001, Student-Newman-Keuls test followed by one-way ANOVA.
